# Environmental awareness and guests’ intention to visit green hotels: The mediation role of consumption values

**DOI:** 10.1371/journal.pone.0248815

**Published:** 2021-05-06

**Authors:** Mustafa Demir, Husam Rjoub, Mehmet Yesiltas

**Affiliations:** 1 Department of Business Administration, Cyprus International University, Mersin, Turkey; 2 Department of Accounting and Finance, Faculty of Economics and Administrative Sciences, Cyprus International University, Mersin, Turkey; 3 Faculty of Economics and Administrative Sciences, Cyprus International University, Mersin, Turkey; Institute for Advanced Sustainability Studies, GERMANY

## Abstract

The paper aimed at examining the influence of consumer’s environmental awareness on their intention to visit green hotels in north Cyprus, the mediation role of consumption values. Research on eco-friendly hotels stressed that conventional hotels do not make available to consumers the environmental awareness and consumption values, whereas, there is possibility that these benefits might not sit well with the hotel potential consumers. Aside highlighting the features of green hotels, it is imperative to examine how consumers perceive consumption values. A questionnaire-based survey was used to examine the study’s objectives. A total of 400 customers at 20 five-stars hotels that were randomly selected across the 3 big cities in north Cyprus (Girne, Lefkosa, and Magusa) were approached and invited to participate. The structural model was tested using structural equation modeling (SEM). The configuration model was analyzed using the partial least square (PLS) method. The finding from the SEM results shows that environmental concern directly and positively influenced the guests’ intentions to visit hotels in north Cyprus. Moreover, functional and emotional values were found to mediate the relationship between environmental concern and knowledge on the guests’ intention to visit north Cyprus hotels. Finally, the study suggest that the research will be of benefit to the managers of hotels in north Cyprus to know the significance of developing consumer’s green awareness and to market it to the customers.

## 1. Introduction

Moyle, Croy, & Weiler [1] in their study opined that the world comprises of well over 100,000 islands that accommodates over 400 million people. In such island which north Cyprus is among, tourism has been the economy bedrock of such country [2]. In support of the tourism industry, hotel services needs to be developed to cater for the accommodation of tourists. Hotel service being a service-oriented industry is believed to contribute to the environmental pollution [3]. Meanwhile, it was suggested in the literature that hotel sector generates quantum of waste and significant volume of water, energy and other natural resources are consumed [4, 5]. In the efforts of the hotels in ensuring an effective service to their guest, their service could cause a severe influence on the environment.

In the recent time, the issue of sustainable development is gaining more attention at all levels and in all economic sector, most especially the tourism and hotel industry [3, 6–8]. Most of the countries where tourism contributes a significant percentage to their GDP, efforts in ensuring the sustainability of their environment to keep track with the market demand is important [3, 9, 10]. Northern Cyprus is one of such countries, which makes this study to be worthy.

The demand for green hotels in recent time has been growing. Norazah [11] noted that because of the increase in people’s understanding of their environment, the consumers’ ecological attitude in making decision, the awareness and green goods and services acceptability are greatly influenced. Though the consumer with knowledge of green hotel understand that lodging in such hotel is expensive, however the cost does not deter them in staying in green hotel because they are interested in bearing cost of any products and services that are harmless to the environment [12, 13]. The decision of the consumer is based on their believe that they are making contribution to reduce the environmental pollution to make it safe for the unborn generation [2, 13, 14].

The research on eco-friendly hotels has been gaining more attention among the researchers. Lots of studies have been carried out to understand better the guest intention on visiting green hotels. Among the previous studies are [4, 15–24], who have examined the individuals’ intention to visit green hotels and the factors that motivate them. Meanwhile, some of the researches were carried out in the developed countries, or in some few developing countries [17, 22]. Few or no research has been carried out to understand the intention of north Cyprus hotels visitors on visiting green hotels. Moreover, the research in north Cyprus may be different from other developing and Island countries owing to its cultural differences and international diplomatic issues. Some of the previous literatures in the demands of green hotels by the customers are majorly on the guests towards green characteristics [12, 20, 22, 25, 26]. But in the literature, evidence abounds that hotels guest attitude towards eco-friendly products/services were in most cases not influenced their buying attitude [17, 27, 28]. While some customers feel the conservation of the environmental should be government business, some believes that there exist a trade-off between long-run social rewards and individual needs in the short-run. Goldstein et al. [26] noted in their study that some hotel visitors wonder what would be their reward if green practices assist the hotels to have minimal operational cost. So the point here is that if the guests are aware that the green hotel will benefit them or assist in certain personal goal achievement, there is tendency that the visitors will be more involved, and thus a more product positive assessment will be achieved.

Suffice to say that the green hotels consumers’ clear behavioral change is necessary to be examined from this angle, in a case where consumption benefit is conceived to shape their attitudes. Most especially the perceived consumption value of what will be gained and given, also its contribution in determining intention to purchase green products has not been thoroughly dealt with in the literature. Meanwhile, Lee et al. [29] and Barber [30] mentioned guest’s expected result of lodging in a green hotels, and what the guests have to offer or sacrifice, are factors that could be significant in green products, but yet to be fully addressed. Thus, it if evident in the literature that previous studies in this area have not exhaustively investigate the effect of the consumption value of the tourist in the nexus between the tourist environmental knowledge and awareness and their intention of visiting a green hotel, which we believe is important owing to its possible aiding of the tourist understanding of hotel innovation contribution to the sustainable development [17, 20]. Therefore, the observed gap in the literature will be addressed in this study and contribute significantly to the literature on green hotels, especially in north Cyprus which has not been previously investigated.

In addressing the issue raised in this study, social exchange theory (SET) will be employed as the theoretical framework. According to Emerson [31], SET enable researcher to assess the multi-faceted dimensions of benefits considers by the customers to achieve and sacrifice at every stays at green hotels, and what to add to enhance researcher prediction of purchase intention determinants. Zeithaml [32] argued further that the process of making decision by the consumers is impacted upon by the guest personal values and that the knowledge and awareness of certain factors by the consumers will influence the perceived consumption value formation. Reason being that the statistical proofs has shown a significant relationship between environmental concern (EC) and guest intention to purchase green product/ services [30], so also the contribution of EC to assist in formation of green product related perceived consumption values. This paper is aimed to enlarge Social exchange theory by adding environmental concern (EC) as an independent variable in the framework and perceived consumption values as a mediator variable to provide a clear understanding of how these variables influence the decision making process of the visitor to the green hotels.

## 2. Conceptual framework and research hypotheses

### 2.1. The basic idea of social exchange theory (SET)

In a general term, SET proposed that an individual is ready to involve in an exchange of goods/services if the person knows there is a benefit from it, and such benefits that will accrue will be greater than costs he/she is putting in the exchange [33]. Subsequent development on the theory was evident in the work of Homan [34, 35], as a marginal utility theory, to Blau [36] as a sociological instrument, and later Emerson [37] as a psychological instrument. The works of these authors has a semblance of same ideas about the theory. They all viewed SET that the attitude of individual in the exchange lies solely on the rationality of the magnitude of the benefit he/she will get, and that the exchange should be able to provide rewards in returns. This implies that exchange rewards between the stakeholders should be handled fairly for the interaction to take place. Moreover, the stakeholders will subsequently make efforts to maximize the benefits from the interaction, so also to be certain that expenses on the exchange will not be more than the rewards, and lastly, the exchange should be well understood by both actors and devoid of coercion.

SET has been found to be a general theory that could be employed in several disciplines such as sociology, psychology, economic, management, and anthropology. In 1961, Homan employed a framework of SET based on attitudinal psychology. The study argued that SET must lay emphasis on physical interaction; it should also center on direct and restricted exchange between people. Meanwhile, the study shed more light on how stakeholders let loose the actions depending on the quantity of the value for him/her. In the study of Blau [36], SET was employed by borrowing economic approach. The study argued that exchange relationship involves activities putting together contingents on feedbacks that will be beneficial to both parties; argued further that the activities will stopped once the expected feedback from the exchange are not at advantageous to the actors. The psychologist opined that Homan [35] study is suitable for attitudinal explanation.

Meanwhile, in tourism research, the theory was utilized many years back. The theory has a potential in tourism industry, as it shows that the economic, environmental, and socio-cultural influence on tourism could be explained with the theory. From this perspective, changing the community native attitude of consumption stand for a cost, as negative cultural influence leads to development of tourism, in order words, it is safe to conclude that the rewards and cost do not have to do with economic side. In the study of Gursoy et al. [38], economic, environmental, and social influence were discussed and the study analyzed their impact differently on the native perception, and different findings were revealed in the study based on individual type of influence. SET has been widely used in previous studies for the analysis of tourist perception and the local community towards tourism development [39, 40]. The studies have been in relation to the perception of these stakeholders towards the environmental, economic, and sociocultural effect of tourism development. In other words, SET has assisted various researchers in this field to understand the responses of local communities and tourists towards the significance of future societal and tourism development like green tourism [39, 41–43]. Some studies observed that aside from the previous benefit of SET, it also explain the economic and non-economic benefits from active nexus between the local communities and tourism actors [41, 44]. In conclusion, SET can be employed as a conceptual framework to understand how and reason for negative or positive perception of visitors on the tourism development.

### 2.2. Additional variables and the guests’ visiting intention

#### i. Environmental concern (EC)

Environmental concern is about examination of, or a behavior towards evidence, individual attitude, or other attitude with implication for the natural habitat [45]. People that are environmental conscious tends to know the hazards associated with the environment pollution and provide cooperation in ameliorating or eradicating the problem, and also show willingness to make contribution in solving the problem [46]. The study of Maloney and Ward [47] cited in Jiang and Kim [48] argued that EC could be scaled in many dimension. For instance, the willingness and exact attitude of the people to conserve the environment and, the manner the people interact with such issue emotionally, so also their environmental knowledge. Meanwhile, eco-friendly behavior is multiple dimensional [13], previous research on eco-friendly hotels have often examined it from multi-dimensional view. In line with these, our study utilized the generally used factors emanated from previous studies to provide a statistically proved green behavior of hotel consumers.

#### ii. Environmental knowledge

Alba and Hutchinson [49] opined that in consumers’ studies, knowledge is believed to have impact on the process of decision making, and the way at which the customer’s assess the product/services. In terms of environmental knowledge, it was referred to as the consumer’s ability to distinguish a number of environmental related signs, concepts and attitude. Differently from global knowledge of customers’ fundamental knowledge of natural world, Darnall et al. [50] opined that there should be other case of environmental understanding that is predicated on action. This includes an awareness of the implication of one’s activities on the environment and of the solution that can enhance attitude. Thus, it is believed that there is possibility that consumers’ with a global environmental understanding will understand with ease the green activities in green hotels. Because of their understanding of the environmental responsibility of both actors as critical and how they carry out their eco-friendly practices, the consumers will be aware of the implication of lodging in the conventional hotel and therefore opt to lodge at a eco-friendly hotel.

#### iii. Perceived consumption value

Zeithaml et al. [32] viewed perceived consumption value (PCV) as an assessment by the consumers of the equivalence between rewards and cost. In a nutshell, it stands for a compromise of the critical “give” and “take” elements. Prior definitions only conceived benefits as a compromise between price and quality, which was argued by many scholars that the definition should, encompasses a wider and globally acceptable idea that will enable the value to be scaled in a multi-dimensional way [51–54]. In response to these, there was a development of a five-dimension model that includes functional, emotional, social, epistemic, and conditional value. Because of the relation of epistemic value to a new idea part of a good, and provisional benefits came up when some unexpected things like illness occur, modification to the model was carried out by Sweeney & Soutar [54] who came up with three-dimensional model which are functional value (quality and price), social, and emotional value. The study further suggested that owing to the influence of quality and price on consumer’s attitude, the two dimensions could be scaled differently.

The study of Koller et al. [51] noted that the green car consumption comes with perceived emotional, functional, and social value. In a not recent study by Fu and Hu [55] where they investigated the influence of low-carbon usage in an eco-friendly hotel on the manner in which the guests sensed the consumption benefits, while Barber [30]) aimed to examine the benefits derived by consumers for lodging in a green hotel. In these studies, there are shortcomings of employing this model. On one aspect, these studies focus more attention on the gains of customers, instead of what to offer for the exchange. Despite the fact that the consumers are considered to have received both financial and non-financial costs [54, 56]. Meanwhile, Sanches et al. [57] argued that the subsisting understanding of value was incomplete with a well-documented “cost” measure, most especially in green hotel. In this case, the attribute of ‘price’ was removed from the initial framework and now measured with a multiple dimension, together with other units of PCV. In other words, utilizing green hotel would be a new feel to the consumers; their mental ability to fulfill a desire for understanding should not be overlooked. Therefore in this study, the modified model by Sweeney and Soutar [54] was employed. Moreso that consumer’s perceived consumption value occur in the entire process of consumption [58], and consumers assess the service provided prior to making the purchase [59], in other words, perceived consumption value can be developed in the absence of goods/services being purchased or utilized. This research therefore explore the consumer’s perceived consumption value before the purchase is made to understand what value do they think will be accrued to them and how it influence their decision to stay in a green hotel. In line with this suggestion, rewards of eco-friendly product/services could be in form of health, contribute to public product, and social or emotional rewards [60, 61].

*Functional value*. It is generally proposed to be developed by quality and price, and to be scaled differently because quality is found to have positive impact on the consumption value, while price is found to have negative impact [54]. In line with the objective of this study, quality and price is utilized to be stand for functional value, while functional value is included as one of the dimensions of the multi-dimension measure of perceived consumption value (PCV). The utility achieved from the perceived and expected functioning of the product/service is refers to as the quality- based functional value [54]. Kim *et al*. [62] and Lee *et al*. [29] in their studies opined that in relation to the hotel industry, the utilization of non-toxic stuffs, disposing of plastic instruments with glassware or show-case of natural food, will not only limit the environmental pollution, but will as well enable the consumers to have an eco-friendly environment that will make them to have a safe and healthy experience.

*Social values (SV)*. Sweeney and Soutar [54] described SV as the usefulness accrued from a product’s effort to improve social self-concept. In the study of Maibach [63], it was argued that consumers’ would be encouraged if their efforts in contributing to ameliorating environmental challenges are recognized by others. The form of automatic expression feelings that consumers want to show their understanding of the environment and anticipate an acknowledgement from the public [28]. In the same vein, Barber [30] was of the opinion that guests lodging at green hotel believes that their option of staying there will influence other guest to the hotel.

*Emotional values (EV)*. This is described as the utility gained from the impression that a good bring forth [54]. Because of the harmonious performance of green consumption with environmental conservation, it often brings forth positive emotions [64]. The feelings of good living are associated with altruism, which Jeong and Jang [65] said to be good things for others with no intention of getting anything in return. A developing want for an enhanced good living as came out as a significant rewards from feelings am0ng the hotel consumers [66]. According to Hotel online [67], the consumers believe that their decision to purchase is a determinant factor in protecting the environment and bequeath a better green natural habitat for the unborn generation.

#### iv. Attitudinal intention

In the study of Ajzen [68] and Lee et al. [14], attitudinal intention of consumers was defined as the acknowledged possibility of performing a particular attitude. Most importantly in the green hotel context, it was described by Lee et al. [29] as the aim of buying good products/service and suggests it to other people. Jeong & Jang [65] employed this term to measure a situation of possibility of the consumers to re-visit green restaurants and aim to suggest it to someone else. In respect of this study which among the aim is to explore consumer’s behavior towards lodging at green hotel, which is possible for the consumer to have had previous experience, their aim to suggest is however not included. Thus, since findings from previous studies indicate a significance relationship between AIs and actual attitude, it makes sense to examine consumers purchase intention and distinguish its determinant factors. In recent years, greening has been trending in the hotel industry. It is believes that it will not only minimize the environmental pollution, but it will also assist hotel managers to have cost-saving on their operational cost and these will improve promotion of their hotel [17, 69, 70]. In some case, the hotels that utilized large quantity of energy, natural resources and non-recyclable product are believed to be a significant contributor to environmental pollution [17]. Meanwhile, paying no adequate attention to the resources saving and protecting the environment could be harmful [71], however, there is growing concern on how to ameliorating the resources consumption and emission reduction, and to join hands with the community to ensure a safe environment. Lee et al. [14] noted that another factor of adopting green management as a strategic instrument for improving the competitive advantage of the hotels is as a result of customers” demand. The more the customers increase in awareness about the environment in the hotel industry, so also their demand for green hotels is becoming evident [72].

According to Butler [73] in his study on the North America hotel, it shows in the study that 75 percent of the guests to the hotel are interested in being a partaker in environmentally favorable program. In support of Butler [73], Lee *et al*. [14] noted that as a result of the customers’ awareness of the environmental issues which are caused by the hotel services, customers are more interested in the hotel that follow through green practices. Looking at the change that is happening in the hotel industry, manager who desires to increases competitiveness of their business through advertisement of green environment products and services is required to have knowledge on the eco-friendly behavior of their guests [29, 74]. Though, the study carried out to identify significant factors that determine the decision making of the consumers in choosing favorable green products and services are scant (Han et al., 2010). Among the questions that remain unanswered are how the consumer environment concern develops, and what factor motivate their desire for green products and services and develop it into action, so also to know whether their decision to visit green hotels is being hindered by some factors.

A green hotel according to Han et al. [12] is described as a hotel that has environmental favorable services that established and abide programs that are ecologically effectual, that is aimed at protecting the planet. Cyprus Tourism Organization (CTO) (media.visitcyznms.com/ups) also described green hotel as those hotels that are aiming to reduce the negative influence their activities could have on the environment, and ensure maximization of their benefits through strengthening of sustainable development programs and other actions that will be less harmful of the environment. In particular, green hotels are dedicated to meet the energy efficient standards, water preservation, green products usage, waste management, management of air quality, regulation of noise pollution, management of toxic waste, human resource management, joining hands with the community organizations and environmental policies that are related to the operation of hotels [2, 3, 75]. Green hotel is more explicitly defined by Green Hotel Association [76] cited in Wang et al. [23] as hotels that are inclined to tailored eco-friendly services by ensuring energy use, natural resources and water efficiency, without being detrimental to the customers’ satisfaction and provision of quality services. Lots of eco-friendly management actions in the hotel industry, for example solid waste reduction, and reuse of indestructible products are incorporated in the definition [14].

Lots of benefits inherent in green hotels, not only that social advantages are obtained and consumers are enlightened about the natural habitat, however it improves the credibility of hotels also, reduce the functionality costs, and appreciable economic rewards are generated. In other words, in the present competition in the hotel industry, green hotels have become a significant strategy for hotels to carve niche for itself in the industry. In the recent time, studies about green hotels in north Cyprus hotel industry are becoming significant. Record shows that most of the 5-star hotels in north Cyprus are implementing eco-friendly hotel [77]. However, some small and medium-sized hotels managers are yet to have adequate understanding and benefits of green hotels, and they failed to tender towards green hotels. In a nutshell, there is more to be done on the hotels in north Cyprus [77]. Meanwhile, it is pertinent to point out that the hotel management should not be solely hold responsible for the unsatisfactory green hotels development; the hotels guest are also a part to the factors responsible for the underdevelopment of eco-friendly hotels, because their intent to lodge at the hotel is important in enhancing green hotels development [4]. Therefore, the transformation of hotels into an eco-friendly one should be the cooperation of both the hoteliers and the guests [78, 79].

### 2.3. Hypotheses developments

#### 2.3.1. Environmental Concern (EC) and Attitudinal Intention (AI)

Lots of research has documented that customers with high degree of environmental concern will tend more to involve in green actions, for instance reuse and energy consumption [12], so also eco-friendly purchase attitude [17, 25, 80–82]. Manaktola and Jauhari [60] study on the hospitality industry found that consumers that are environmentally concerned favors green product purchase. Similarly, Millar and Baloglu [61] hypothesized that a prospective guest with background understanding of environment, has a high level of involvement endurance with green activities. Han et al. [12] and Jiang & Kim [48] also indicated in their studies that a consumer who performs more environmental friendly activities has high probability of choosing green hotels.

Among the recent studies is the study of Bauer, Arnold, & Kremer [81] and Ibnou-Laarousi, Rjoub, & Wong [17] who found a positive nexus between EC and guest intention to visit green hotels. In references to several conclusions in the literature, we considered a direct and positive effect of EC on guest intention to visit green hotels.

Thus we proposed that:

*H1*. *Environmental concerns have a direct and positive influence on guest intention to visit green hotel*
*H2*. *Environmental knowledge has a direct and positive influence on guest intention to visit green hotel*

Though, environmental behavior is significant not only because it has impact on the consumers’ behavior, but because it also provide proofs of a significant environmental ethics that guide what rewards environmental friendly seeks [83]. McCarty and Shrum [84] cited in Jiang & Kim [48] argued that consumers’ positive behavior towards socially conscious attitude could be impacted by the magnitude of the value the consumer placed on the protection of environment. But the mediation function performed by the consumption value (PCV) on the influence of environmental concern (EC) on consumers’ green hotel visitation has not been thoroughly dealt with in the literature.

In this study, we therefore proposed as follow:

*H3*. *Environmental concerns have a direct and positive impact on (a) functional value*, *(b) social value*, *and (c) emotional value*
*H4*. *Environmental knowledge has a direct and positive impact on (a) functional value*, *(b) social value*, *and (c) emotional value*

#### 2.3.2. Consumption value and attitudinal intention (AI)

In the context of hotel industry, consumers’ perceived consumption value has been identified as a critical factor that influences the attitudinal intention of the consumer [85]. Meanwhile, the contribution in the green hotel industry has been under-studied. Owing to the fact that this value expectation will preponderantly channelized the process of decision making and consumer’s option would be a responsibility of multi-dimension of consumption value. Therefore, in this study, the pre-buying perceived consumption value influences on the guest intention of visiting green hotel will be explored by identifying the role of PCV. In the literature, a perceived reward has been one of the universally acceptable antecedents’ variable in examining tourist attitude [86]. Findings shows that rewards has a clear-cut part that makes differential share in a particular choice making [87]. In the study of Sheth et al. [53], it was found that functional value was central to the positive impact on the choice made by the consumers, and different value attribute is significant, meanwhile, it depends on the type of product that is in consideration.

In green hotel context, emotional value has been proved to be a critical determinant factor of environmental friendly activities, and green product consumption [28, 30, 88]. Some studies posited that people tends to purchase green product/services where information pertaining to green product/services are displayed and the guests are tends to opt for or willing to pay for it [89–91]. Young and Jang [92] examined the role of characteristics of food quality in a green restaurant; healthy option available was found to have a critical impact on behavior intention of the consumers. Thus it is safe to propose that perceived consumption value (PCV) will be a determinant factor in the process of decision as regards their intention to visit green hotel.

*H5*. *(a) Functional value*, *(b) Social value*, *and (c) Emotional value have a direct influence on guest intention to visit green hotel*.

#### 2.3.3. PVC as a mediator

Granzin and Olsen [93] in their study found that consumers with high level of environmental concern is believed to envisaged more rewards from assisting with the environmental conservation. Similarly, Koller et al. [58] in their work documented that the impact of environmental treasure on other value attributes is well articulated for more eco-friendly concerned customers. Differently, general customers estimated only lower price, these customers comprehended emotional value to be of more significant. Thus, eco-friendly hotel consumers are accepted to receive more positive beliefs about eco-friendly hotel will be of benefit to them or society in general and therefore motivated to make a decision in lodging at the green hotel.

*H6*. *Functional value functions as a partial mediator of the influence of (a) environmental concern and (b) environmental knowledge on guest intention to visit green hotel*.
*H7*. *Social value functions as a partial mediator of the influence of (a) environmental concern and (b) environmental knowledge on guest intention to visit green hotel*.
*H8*. *Emotional value functions as a partial mediator of the influence of (a) environmental concern and (b) environmental knowledge on guest intention to visit green hotel*.

## 3. Measure and data collection

This study employed structured questionnaire that was designed in line with the objective of the study. The first section consists of demographic characteristic of respondents such as gender, age, marital status, educational level, family size and employment status. The second section comprised of questions about the perception of the respondents on the environmental concern and environmental knowledge, which was adapted and modified from Jiang and Kim [48]. EC and EK were measured with seven (7) and three (3) items respectively. The third section comprises of questions on the respondents perception on consumption value. The consumption value was measured with three sub-constructs in relation to the guest understanding of an eco-friendly hotel, and these were adapted from Wong & Yeh [94]. The constructs which are emotional value, functional, and social value were measured with three (3), eight (8), and four (4) items respectively. Lastly, the last part comprised of questions on the guests intention to visit green hotels. The guest intention to visit green hotels was measured with three (3) items that were adopted from Cheng et al. [95] (see S1 Appendix). It is suffice to note that each of the construct adapted from previous construct has a cronbach alpha that is greater than the threshold value of 0.70. Five-point likert scale was adopted for the measurement, which ranges from 1 (strongly disagree) to 5 (strongly agree). Twenty 5-stars hotels from three largest cities (Lefkosa, Magusa, and Girne), since they are the ones that engage in ecological solutions composed our population. Data were collected between July 5th, 2019 and August 20th, 2019. The population of our study was (N = 14,364: Source: hotel management) according to the bed capacity in the 5-stars hotel. According to the north Cyprus Hoteliers Association, the study sample data was collected from 20 five-star hotels in north Cyprus as follows: Lefkosa Two, five-stars hotels called, “Grand Pasha Nicosia Hotel Casino Spa” and “Merit Lefkosa Hotel and Casino”, also in Girne twelve, five-stars hotels called, “Elexus Hotel Resort Casino”, “Lord’s Palace”, “Cratos Premium Hotel and Casino and Spa”, “Kaya Palazzo Resort and Casino”, “The colony Hotel”, “Merit Crystal Cove Hotel and Casino”, “Rocks Hotel Casino”, “Acapulco Resort and Convention and Spa Hotel”, “Vuni Palace Hotel”, “Grand Pasha Kyrenia Hotel Casino Spa”, “Malpas Hotel”, “Savoy Hotel” and “Jasmine court Hotel”. In Magusa, we selected six 5-stars hotels called “Salamis Bay Conti Resort Hotel”, “Arkin Palm Beach”, “Limak Cyprus Deluxe Hotel”, “Concorde Luxury Resort and Casino Convention Spa”, “Kaya Artemis Resort Hotel”, “Noah’s Ark Deluxe Hotel & Spa” (http://www.northcyprus.net/). Sample size was calculated based on the given population and found 374 respondents with 5% confidence interval. The study used disproportionate simple random sampling in which 20 questionnaires were handed over to each of the managers of each hotel for onward distribution to their customers. The managers handed the 20 questionnaires to the hotel guest randomly at the reception.

## 4. Data analysis and results

This study employs Partial Least Square (PLS), being regarded as the most fully developed system among the Variance-Based SEM methods [96], and it was carried out in four-steps according to Dijkstra & Henseler [97], which are first to examine the psychometric properties of our construct; secondly attenuation correction for those constructs; third, parameter estimation; and lastly, bootstrapping for the significance of estimations. Specifically, convergent, discriminant validity and composite reliability are examined through the utilization of partial least square (PLS) algorithm [98]. Our research model was analyzed through the utilization of structural equation modeling, while the significance of the model and the evaluation of the significance of mediating impact was analyzed with bootstrapping [99], size effect (f^2^) and standardized root mean square residual (SRMR) was generally utilized to evaluate the fitness of the model.

### 4.1. Respondents’ demographic analysis

The demographic analysis of the respondents is presented in Table 1 which reveals that 60.5% of the respondents were male, while 39.5% were female, which is an indication that the respondents are mostly male. In respect of their age, the age group of 35–44 years and 45–54 years were found to dominate the respondents with 39.5% and 29.8% respectively. The analysis of the respondents’ marital status shows that 55.8% were married, while 28.5% and 15.8% were divorced/widowed and single respectively. Analysis shows that most of the respondents are learned with 50.5% of them having diploma, while 37.3% having Bachelor degree. Moreover, about 67.8% and 23.8% of the respondents have a family size of 2–3 persons and 4–5 persons respectively. In addition, the business people and respondents employed on full time have the largest percentage with 32% and 29% respectively. Lastly, the descriptive statistic of the constructs as presented in Table 2 shows that the mean and standard deviation of “Environmental Concern” (EC) were 3.43 and 0.54 respectively which is an indication of a neutral to average concern. “Environmental Knowledge” (EK) was 4.03 and 0.50, demonstrating moderate to average knowledge, emotional value (4.00 and 0.50), functional value (3.43 and 0.52), social value (4.00 and 0.60), intention (4.15 and 0.69) indicating a moderate to average to moderate perception.

**Table 1:** Respondents’ demographic analysis.

		Frequency	Percent
Gender	Female	158	39.5
	Male	242	60.5
Age	18–24 years	20	5.0
	25–34 years	51	12.8
	35–44 year	158	39.5
	45–54 years	119	29.8
	55–64 years	41	10.25
	65 years and above	11	2.75
Marital status	Single	63	15.8
	Married	223	55.8
	Divorced/Widowed	114	28.5
Level of Education	High school	32	8.0
	Diploma	202	50.5
	Bachelor’s degree	149	37.3
	Master’s degree	10	2.5
	Doctoral degree	7	1.75
Family size	1 person	18	4.5
	2–3 persons	271	67.8
	4–5 persons	95	23.8
	More than 5 persons	16	4.0
Employment status	Student	31	7.7
	Housewife	69	17.3
	Unemployed	10	2.5
	Business	128	32.0
	Full-time	116	29.0
	Part-time	46	11.5

**Table 2:** Constructs descriptive analysis: Mean and standard deviation.

Constructs	Mean	Standard Deviation
Environmental Concern	3.43	0.54
Environmental knowledge	4.03	0.50
Emotional value	4.00	0.50
Functional value	3.43	0.53
Social value	4.00	0.60
Guest intention	4.15	0.69

### 4.2. Measurement model testing

The psychometrics properties of the variables were analyzed and the result presented in Table 3, and the model fit statistics shows that the model measurement of data was sufficient. The result from the table shows that the loadings were significant as none of the loadings has a value that is less that threshold of 0.70 [99]. Meanwhile, in order to assess the validity of the construct, so as to ensure the uni-dimensionality of the factors, a characteristic that is being examined through convergent validity. The dominant measure of convergent validity is the average variance extracted (AVE), the result as presented in Table 3 reveals that EC, EK, EV, FV, GI, and SV has a AVE value of 0.58, 0.80, 0.67, 0.65, 0.86, and 0.76 respectively. It is clear from the result presented that none of the value is less than 0.5 which is regarded as the acceptable minimum threshold [96]. As for the measurement of the total value of true score variance in relative to the total scale score variance, the result as presented in Table 3 shows that the composite reliability (CR) value for all the variables are greater than 0.80. Though. Brunner & Sub [100]) opined that the range of threshold among the researcher for CR is still a moot topic, but our values are greater than 0.6 as suggested by Hair et al. [101], therefore our result shows that the latent variables has a reliable internal consistency.

**Table 3:** Validity and reliability for construct.

Latent variable	Indicator	Loadings	AVE	CR	VIF
Environmental Concern (EC)	EC1	0.791	0.58	0.906	2.08
	EC2	0.739			1.92
	EC3	0.822			2.70
	EC4	0.768			1.87
	EC5	0.78			2.52
	EC6	0.793			2.09
	EC7	0.72			1.45
Environmental knowledge (EK)	EK1	0.913	0.80	0.922	2.70
	EK2	0.858			2.01
	EK3	0.907			2.62
Emotional value (EV)	EV1	0.752	0.67	0.857	1.23
	EV2	0.854			2.06
	EV3	0.84			1.99
Functional value (FV)	FVP1	0.783	0.65	0.937	2.39
	FVP2	0.593			1.53
	FVP3	0.817			2.64
	FVP4	0.74			1.96
	FVQ1	0.893			4.68
	FVQ2	0.877			4.33
	FVQ3	0.904			3.92
	FVQ4	0.813			3.57
Guest intention (GI)	INT1	0.916	0.86	0.948	3.03
	INT2	0.951			4.53
	INT3	0.913			3.26
Social value (SV)	SV1	0.876	0.76	0.926	2.78
	SV2	0.913			3.37
	SV3	0.87			2.50
	SV4	0.824			2.16
Model fit statistic: SRMR = 0.07, X^2^ = 1864.78, NFI = 0.78, rms Theta = 0.16					

Note: AVE = Average variance extracted; CR = Composite reliability; SRMR = Standardized root mean square residual; X2 = Chi-square; NFI = Normed fit index; rms Theta = Root mean square error correlation; VIF = Variance inflation factor.

Moreover, in order to examine if all the ingredients contribute significantly and substantially, the sign and the magnitude of the indicator weights as well as their significance were evaluated by assessing variance inflation factor (VIF). The result as presented in the Table 1 shows that the items has VIF ratio that ranges between 1.23 and 4.68. This implies that the result is in consistent with Hensele, Hubona, & Ray [96], who suggested that value not less than 1 and greater than 5 is considered to be ok.

To check the discriminant validity of the variables, Fornell-Larcker criterion as proposed by Fornell and Larcker [102] and Heterotrait-Monotrait Ratio (HTMT) by Henseler et al. [103] was employed. While Fornell-Larcker criterion says that a factor’s AVE should be higher than its squared correlations with all other factors in the model, Henseler et al. suggests that the HTMT ratio should be significantly smaller than one. The result as presented in Table 4 shows that latent variable EK’s AVE is found to be 0.80, thus, its square root becomes 0.89. This value is greater than those in the column of EK (0.60, 0.64. 0.55, 0.62), and also greater than those is the row of EK (0.80). The results for other variables is similar to that of EK, which shows that our factors discriminant validity is ok and in consistent with Fornell and Larcker [102]). Moreover, the HTMT ration as presented in Table 5 indicate that none of the latent variable has a ration that is greater than 1 as suggested by Henseler et al. [103] Lastly, in other to ensure there is absence of “common method bias (CMB)” in our measurement, we first employed Harman’s one-factor test for the assessment of the common method variance in accordance with Podasakoff et al. [104]. In this case, “principal component analysis (PCA)” was carried out and the result revealed that there was no dominant by a single factor. But due to the criticism of this method, the suggestion of Kock [105] was followed to examine the VIF. Kock [105] suggested that in PLS-SEM analysis, the VIF can be examined to check for the presence of CMB. Hence, our result that is presented in Table 3 revealed that none of the VIF value is less than 1 and greater than 5, then it is safe to conclude that our measurement has no common bias method issue.

**Table 4:** Discriminant validity (Fornell-Larcker criterion).

	EC	EK	EV	FV	GI	SV
EC	**0.76**					
EK	0.80	**0.89**				
EV	0.59	0.60	**0.82**			
FV	0.66	0.64	0.67	**0.81**		
GI	0.56	0.55	0.61	0.58	**0.92**	
SV	0.64	0.62	0.65	0.70	0.52	**0.87**

Note: (a). EC = Environmental Concern; EK = Environmental knowledge; EV = Emotional value; FV = Functional value; GI = Guest intention; SV = Social value.

(b) The square root of AVE of every multi-item construct is shown on the main diagonal.

**Table 5:** Heterotrait-Monotrait Ratio (HTMT).

	EC	EK	EV	FV	GI	SV
EC						
EK	0.918					
EV	0.723	0.734				
FV	0.73	0.707	0.793			
GI	0.621	0.616	0.727	0.62		
SV	0.723	0.694	0.792	0.761	0.575	

Note: EC = Environmental Concern; EK = Environmental knowledge; EV = Emotional value; FV = Functional value; GI = Guest intention; SV = Social value.

The result presented in Table 6 shows the f^2^ that measure the significant effects of the latent variable. According to Jiang and Kim [48], it makes sense to quantify how substantial they are, which can be accomplished by assessing their effect size. Jiang and Kim [48] suggested that f^2^ values above 0.35, 0.15, and 0.02 can be regarded as strong, moderate, and weak, respectively. Our result as presented in Table 6 reveals that EC, EK, and SV has a very weak effect size on GI, FV can be consider to be weak on GI, while EC and EK has a moderate effect on EV.

**Table 6:** Effect size (*F* square).

	EC	EK	EV	FV	GI	SV
EC			0.059	0.115	0.012	0.106
EK			0.068	0.066	0.008	0.054
EV					0.082	
FV					0.026	
GI						
SV					0.001	

Note: EC = Environmental Concern; EK = Environmental knowledge; EV = Emotional value; FV = Functional value; GI = Guest intention; SV = Social value.

### 4.3. Structural model testing and result analysis

**Fig 1:**
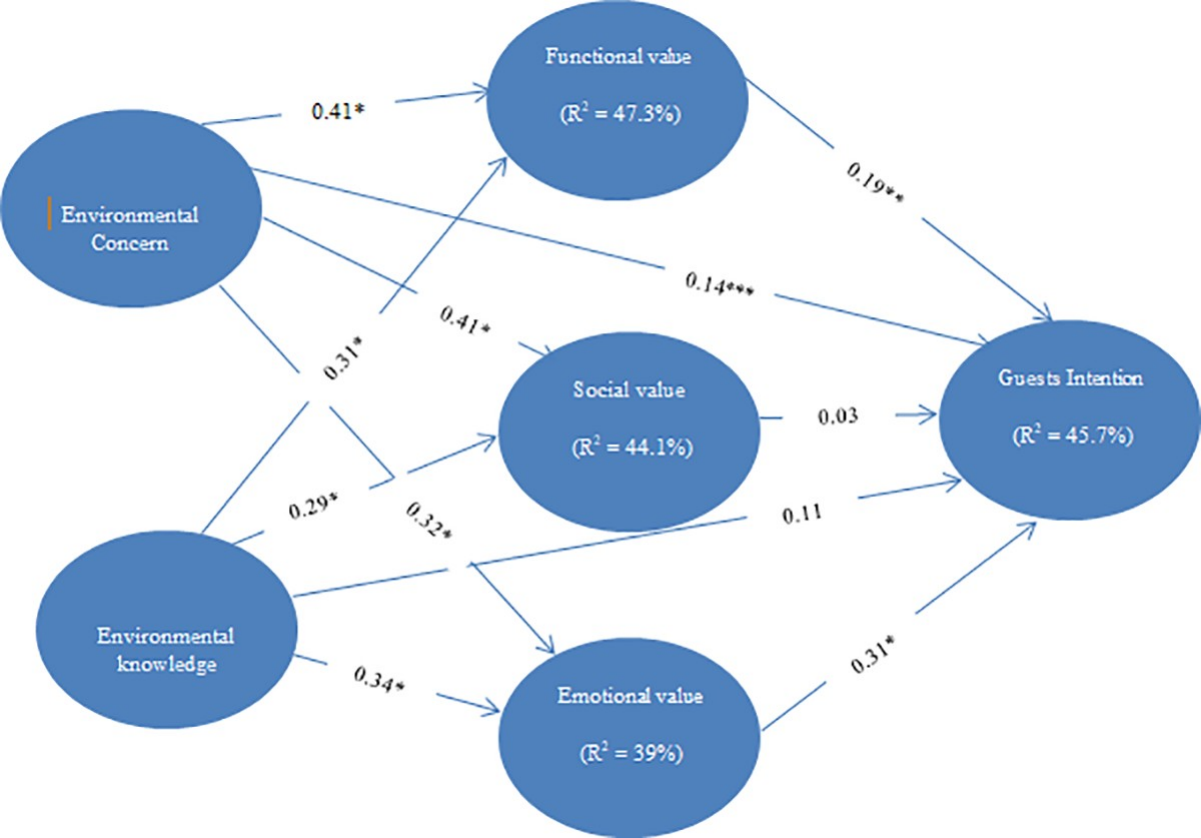
Structural model results.

This study utilized Smart PLS 3.0 to examine the structural model and hypotheses. To examine the sufficient quality of the measurement model, the model was evaluated by bootstrapping with 5000 sample size as suggested by Hensele, Hubona & Ray [96]. The result is presented and depicted in Table 7 and Fig 1 respectively. The adjusted R square in Fig 1 indicates the explanatory power of the predictor variable(s) on the respective construct. The environmental concern and environmental knowledge explains about 47% of guests’ perception on functional value (R^2^ = 0.473); explains about 44% of guests’ perception on social value (R^2^ = 0.441); and, explains about 39% guests’ perception on emotional value (R^2^ = 0.39). Meanwhile, functional value, social value, and emotional value predict about 45% of guests’ intention to visit green hotel (R^2^ = 0.457). In line with the model validity by Chin et al. [106]) who classified the endogenous latent variables as significant, moderate or weak based on the R^2^ value of 0.67, 0.33, and 0.19 respectively. The environmental concern and knowledge contribution to functional value (47%), social value (44%), and emotional value (39%) can be classified as moderate, while contributions of functional value, social value, and emotional value to guests’ intention (46%) can as well be described as moderate.

**Table 7:** Hypothesis testing.

Hypotheses		Beta	*t* value	Decision
H1	EC -> GI	0.41	1.719***	Supported
H2	EK -> GI	0.11	1.23	Not supported
H3a	EC -> FV	0.41	5.322*	Supported
H3b	EC -> SV	0.41	4.352*	Supported
H3c	EC -> EV	0.32	3.237*	Supported
H4a	EK -> FV	0.31	3.767*	Supported
H4b	EK -> SV	0.29	3.058*	Supported
H4c	EK -> EV	0.34	3.554*	Supported
H5a	FV -> GI	0.19	2.145**	Supported
H5b	SV -> GI	0.03	0.38	Not supported
H5c	EV -> GI	0.31	4.226*	Supported
H6a	EC -> FV -> GI	0.078	1.924***	Supported
H6b	EK -> FV -> GI	0.059	1.842***	Supported
H7a	EC -> SV -> GI	0.013	0.364	Not supported
H7b	EK -> SV -> GI	0.009	0.351	Not supported
H8a	EC -> EV -> GI	0.098	2.557**	Supported
H8b	EK -> EV -> GI	0.105	2.666*	Supported

Note: (a) EC = Environmental Concern; EK = Environmental knowledge; EV = Emotional value; FV = Functional value; GI = Guest intention; SV = Social value.

(b) *, **, *** denotes 1%, 5%, and 10% significant level respectively.

Furthermore, the structural model and hypotheses testing results are summarized and presented in Table 7. As presented in the table, it shows that environmental concerns have direct and positive impact on guests’ intention to visit green hotels (*t* = 1.72, *β* = 0.14), therefore hypothesis 1 is supported at 10% confidence level. However, environmental knowledge direct influence on the guests’ intention to visit green hotels was found not to be statistically significant (*t* = 1.23, *β* = 0.11), in this case, hypothesis 2 was not supported. The findings shows further that environmental concern has a direct and positive impact on functional value (*t* = 5.32, *β* = 0.4), social value (*t* = 4.35, *β* = 0.41), and emotional value (*t* = 3.24, *β* = 0.32). Therefore, hypothesis 3a, 3b, and 3c are supported. Moreover, findings shows that environmental knowledge has a positive and direct influence on functional value (*t* = 3.77, *β* = 0.31), social value (*t* = 3.06, *β* = 0.29), and emotional value (*t* = 3.55, *β* = 0.34). Thus, hypotheses 4a, 4b, and 4c are supported. Hypotheses 5a and 5b were accepted since functional value was found to have direct and positive influence on guests’ intention (*t* = 2.15, *β* = 0.19), and emotional value (*t* = 4.23, *β* = 0.31) on guests’ intention to visit green hotel. But hypothesis 5b was not supported as a result of the non-significance of the coefficient (*t* = 0.38, *β* = 0.03).

The mediating influence of functional value, social value and emotional value were analyzed, and the result is presented in Table 7. The findings shows a partial mediating relationship influence for environmental concerns → functional value → guests’ intention (indirect effect = 0.08, *t* = 1.92); environmental knowledge → functional value → guests’ intention (indirect effect = 0.06, *t* = 1.84); environmental concerns → emotional value → guests’ intention (indirect effect = 0.10, *t* = 2.56); and, environmental knowledge → emotional value → guests’ intention (indirect effect = 0.11, *t* = 2.67). Thus, hypotheses, 6a, 6b, 8a, and 8b were supported. However, 7a and 7b were rejected, due to the non-significance of the coefficients.

### 4.4. Summary of findings

The study aimed at formulating and examining research model that evaluate how environmental concerns and environmental knowledge influence the guests’ intention to visit green hotel. The study also examine whether consumption value (functional value, social value, and emotional value), plays a mediating role in the influence of environmental concerns and knowledge on guests’ intention to visit green hotel. Most of the previous researches are mostly concerned with the guests’ behavior as it influences their intention to visit green hotels, relegating the consumption value which created a gap in the literature. Therefore, to fill the gap in the literature, this study investigates the links amongst environmental knowledge and concerns and the guest intention of visiting green hotels. In addition with the role played by the consumption values in the relationship in north Cyprus, a small island state. To the best knowledge of the authors, our study is the first that attempt to investigate this aspect in the green hotels studies in reference to guest understanding of environment and their concerns.

For a better understanding, we applied SET in our study to investigate the effect of consumption values (functional value, social value, and emotional value) as it affects the relationship between guests’ environmental concern and knowledge and their intention to visit green hotels. Firstly, all the hypotheses were conceptualized based on the foundations of the literature to get more robust knowledge of guests’ perception towards green hotels in respect of their concern and knowledge about environment. On one hand, EC is directly linked to guests’ intention to visit green hotels. On the other hands, EK is directly linked to consumers’ intention to visit green hotels. Secondly, guests’ perception on consumption value was investigated using three dimensions (functional value, social value, and emotional value), and conceptualized each of them to mediate the direct relationship between EC and guest intention to visit green hotels on one hand, and the other hand, the direct nexus between EK and guests’ intention to visit green hotels. Therefore, these relationships examine guest’s environmental sustainability attitude towards the contribution of green hotels to sustainable development. We then provide empirical support to justify all the developed hypotheses in this study. Owing to the significance of SET, since it has been widely utilized in various studies to illustrate the economic and non-economic gains by customers for engaging in eco-friendly practices, especially green tourism [39, 41–43, 88], consequently, it was employed in this study to understand the mediating effect of consumption values in the relationship between EC and guest intention to visit green hotels on one hand; and the relationship between EK and guests’ intention to visit green hotels on the other hand. Meanwhile, not all the hypotheses were supported, the findings from this study nevertheless contributes to literature on the relationship between environmental awareness and guests’ intention to visit green hotel.

#### 4.4.1. Theoretical implication

The finding from this study indicates that an environmental concern was a predictor of guests’ intention to visit green hotels. The result is in contrast to the study of Han et al. [74], and Jiang & Kim [48] who did similar study and found no relationship, but in consistent with Manaktola and Jauhari [60], and Milla & Baloglu [61] who concluded in their studies that customer’s with environmental knowledge background is likely to choose green hotel. The results reveals further that environmental concern did not exert influence on functional, social, and emotional value. This implies that the customers’ perception on what they are given and getting in returns for being eco-friendly will be influenced by the guest concern about the environment. Moreover, the environmental knowledge that shows no direct influence on guests’ intention to visit green hotels was found to have direct and positive influence on the customers’ perception value (functional, social, and emotion). In other words, customers’ perception on the ‘give and take’ on environmental issue will be significantly influenced by their knowledge about the environment.

An interesting finding from this study was the multiple categorization of consumption value that was incorporated in the model as mediator in the environmental awareness and guests’ intention to visit green hotels relationships. This is in consistent with previous research [29, 30]. Significantly, the findings from this study suggest that functional value and emotional value have a direct influence on guests’ intention to visit green hotel. This is in consistent with the study of Hartman et al. [107]; Teng [21], and corroborated with the study of Jiang & Kim [48]. Moreover, functional and emotional values were found to be a significant mediator in explaining the relationship between environmental concern and knowledge, and guests’ intention to visit green hotel. The implication of this findings is that eco-conscious consumers will decide to visit green hotel when the functional value is considered to be fair enough.

#### 4.4.2. Management implication

The findings from this study provide a clear indication that the managements of hotels in north Cyprus should communicate more awareness about environmental issues. To make the consumers understand the significant contribution of hotel industry to the environmental conservation. This information provision to the consumers would evoke customer’s feelings towards green hotels. Moreover, the customers’ needs to be studied by the managers, to understand the aspect of green environment that will be beneficial to the prospective customers, before going to them for discussion. More emphasis needs to be placed on the effective way to transmit the information on green practices value to the customer, so as to awaken their concern instead of their feelings about the environmental issues.

In line with the findings of this study, hotel managers should empathize that conveying the information on the functional benefits of a green hotel to the hotel customers would assist in generating a favorable customers’ perception and their intentions to visit green hotels. As a result of the increase in the number of people that are environmentally conscious, the practitioners in the hotel industry should include emotional value into their campaign, so as to complement the functional value-based strategy. Based on the findings from this study, it is pertinent for the hotel management in north Cyprus to maximize the advantage of social media to convey the information about environmental knowledge, the benefits of being an eco-friendly person, and also on what they will experience when staying in green hotels. In doing these, the hotel management should create a system that will enable them to have a feedback from the customers. This will assist the managers to understand the perception and true evaluation of the customers stays in the green hotel. In addition, this study will help hotel managers and owners to understand the requirements of transforming the hotels in Cyprus into green hotel status in order to provide competitive advantage.

### 4.5. Limitations and suggestion for future research

Though, this study contributes significantly to the literature, however it is not devoid of limitations. Firstly, the limitation lies in the form of data collection which was collected solely from the customers that lodge in five-star hotels in north Cyprus, this could to some extent impose a limit to the generalization of the findings. Thus, future studies should utilize this model on customers that lodged in real green hotels. In addition, comparative studies on the customers lodged in conventional hotel and green hotel would be an interesting area to explore in the future. Secondly, this study did not control for the demographic characteristics of the customer, this could to some extent affect the perception of the customers on the subject matter. Therefore, future studies can explore the guests’ demographic factors as moderating variables in the model developed in this study.

## 5. Conclusion

Though studies abounds on the customers’ intention to purchase and repurchase green products based on their consciousness of the environment, but the decision making process of visiting green hotel have not been thoroughly dealt with empirically in the literature. This study examined the relationship between consumers’ environmental concern and knowledge on how it affects their intention to visit green hotels. Moreover, the multi-dimension of consumption values was developed to examine its role in mediating the relationship between environmental awareness and guests’ intention to visit green hotel. Thus, the result contributes to the literature theoretically on the green hotel and customers’ consumption value.

Implication from this study shows that environmental concern performs a significant function in the guests’ intention to visit green hotel, more importantly, the mediating function of functional and emotional value as it mediates the relationship between environment awareness and the guests’ intention to visit green hotel. Therefore, it is pertinent for the hotel managers and marketers to know how to convey the information on the environmental issue to the consumers, so as to arouse their interest in green hotels. In other words, the significant contribution of this research to the literature calls for practitioners to adopt managerial strategies that are equivalent to green management operations. Hence, the greater sustainability measures should be taken and considered in practice, in such a way that the hotel managers and the tourism industry in general will be contributing to environmental degradation reduction when targeting the appropriate ways to amend their practice based on a greener structure.

In conclusion, thus study examined the mediating role of perceived consumption values in the relationship between environmental awareness and guests’ intention to visit green hotels. It will be of interest for the future research to include brand level in the model to understand if the perception of consumers’ value in the economy unit of green hotel will be the same with perception of customers of upscale green hotels.

## Supporting information

S1 Appendix (DOCX)

S1 Dataset (XLSX)
