# Supplementary material for: Environmental awareness and guests’ intention to visit green hotels: The mediation role of consumption values
Source: PLoS One. 2021 May 6;16(5):e0248815. doi: 10.1371/journal.pone.0248815 (PMC8101960; doi:10.1371/journal.pone.0248815)
Supplement: S1 Appendix — (DOCX) [file pone.0248815.s001.docx]

**S1 Appendix:**

| Dear Participant,  The researcher is currently conducting a scientific study aimed at testing the impact of environmental awareness on guests’ intention to visit green hotels in North Cyprus: A field study on Current International travelers’ in the Turkish of Republic Northern Cyprus. As part of the requirements for a Ph.D in Business Administration, and where we entrust you with the constant interest and willingness to support scientific research that serves and develops the community.  We hope that you will be able to read the contents of the questionnaire and choose the appropriate answer for all the questionnaire questions from your point of view. Please note that the information collected will be for academic purposes only and all answers are anonymous and confidential.Our respect. |
| --- |

**SECTION I**

1. **Gender :**

**( )** Woman **( )** Men

1. **Age :**

**( )** 18-24 age **( )** 25-34 age **( )** 35-44 age **( )** 45- age

**( )** 38-42 age **( )** 43 age and/or over

1. **Your Marital Status :**

**( )** Single **( )** Married ( ) Divorced/Widowed

1. **Your Educatıonal Status:**

**( )** High School **( )** Diploma () **( )**  Bachelor’s degree **( )** Master’s degree ( ) Doctoral degree

5. Family size

( ) 1 person ( ) 2-3 person ( ) 4-5 person ( ) More than 5 persons

6. Employment status

( ) Student ( ) Houswife ( ) Unemployed ( ) Business ( ) Full-time Job ( ) Part-time Job

**SECTION II**

| Please indicate your level of participation in the following phrases by placing an "X" in front of your choice of expression. | **Strongly Disagree** | **Disagree** | **Neutral** | **Agree** | **Strongly Agree** |
| --- | --- | --- | --- | --- | --- |
| **Environmental concerns** |  |  |  |  |  |
| 1. I make a special effort to buy paper and plastic products that are made from recycled materials. |  |  |  |  |  |
| 2. I have switched products for ecological reasons. |  |  |  |  |  |
| 3. When I have a choice between two equal products, I purchase the one less harmful to other people and the environment. |  |  |  |  |  |
| 4. I have voted for a candidate in an election at least in part because he or she was in favour of strong environmental protection. |  |  |  |  |  |
| 5. I have avoided buying a product because it had potentially harmful environmental effects. |  |  |  |  |  |
| 6. I have read newsletters, magazines or other publications written by environmental groups. |  |  |  |  |  |
| 7. I have signed a petition in support of protecting the environment. |  |  |  |  |  |
| **Environmental knowledge** |  |  |  |  |  |
| 8. I know more about recycling than the average person. |  |  |  |  |  |
| 9. I understand the environmental phrases and symbols on product package. |  |  |  |  |  |
| 10. I am very knowledgeable about environmental issues |  |  |  |  |  |
| **Functional values PRICE ( FVQ)** |  |  |  |  |  |
| 11. It is worth paying for the quality and the service of green hotels |  |  |  |  |  |
| 12. I think green hotels offer value for money. |  |  |  |  |  |
| 13. It is worthwhile choosing green hotels when traveling. |  |  |  |  |  |
| 14. I think it is economical to visit green hotels. |  |  |  |  |  |
| **Functional value QUALITY (FVQ)** |  |  |  |  |  |
| 15. I think green hotels have consistent quality. |  |  |  |  |  |
| 16. I think the products and services of green hotels are well made. |  |  |  |  |  |
| 17. I think green hotels have an acceptable standard of quality. |  |  |  |  |  |

| 18. I think the products and services of green hotels are reliable. |  |  |  |  |  |
| --- | --- | --- | --- | --- | --- |
| **Social value (SV)** |  |  |  |  |  |
| 19. I think visiting green hotels helps me feel accepted by others. |  |  |  |  |  |
| 20. I think visiting green hotels would improve the way I am perceived by others. |  |  |  |  |  |
| 21. I think visiting green hotels makes a good impression of me on other people. |  |  |  |  |  |
| 22. I think visiting green hotels gives me social approval. |  |  |  |  |  |
| **Emotional value (EV)** |  |  |  |  |  |
| 23. I think visiting green hotels instead of conventional hotels would feel like making a good personal contribution to something better. |  |  |  |  |  |
| 24. I think visiting green hotels instead of conventional hotels would feel like the morally right thing to do. |  |  |  |  |  |
| 25. I think visiting green hotels instead of conventional hotels would make me feel like a better person |  |  |  |  |  |
| **Intention** |  |  |  |  |  |
| 26. I am willing to choose a green hotel when traveling. |  |  |  |  |  |
| 27. I plan to choose a green hotel when traveling. |  |  |  |  |  |
| 28. I will make an effort to stay at a green hotel when traveling |  |  |  |  |  |
